# Systems Level Metabolic Phenotype of Methotrexate Administration in the Context of Non-alcoholic Steatohepatitis in the Rat

**DOI:** 10.1093/toxsci/kfu160

**Published:** 2014-08-21

**Authors:** Michael Kyriakides, Rhiannon N. Hardwick, Zhaosheng Jin, Michael J. Goedken, Elaine Holmes, Nathan J. Cherrington, Muireann Coen

**Affiliations:** * Biomolecular Medicine, Division of Computational Systems Medicine, Department of Surgery and Cancer, Imperial College London, London, SW7 2AZ, UK; † Department of Pharmacology and Toxicology, University of Arizona, Tucson, Arizona 85721, USA; ‡ Faculty of Medicine, Imperial College London, London, SW7 2AZ, UK; § Department of Pharmacology and Toxicology, Rutgers University, Piscataway, New Jersey 08854, USA

**Keywords:** methionine-choline deficient diet, liver toxicity, methotrexate, metabonomics, nuclear magnetic resonance spectroscopy, non-alcoholic steatohepatitis

## Abstract

Adverse drug reactions (ADRs) represent a significant clinical challenge with respect to patient morbidity and mortality. We investigated the hepatotoxicity and systems level metabolic phenotype of methotrexate (MTX) in the context of a prevalent liver disease; non-alcoholic steatohepatitis (NASH). A nuclear magnetic resonance spectroscopic-based metabonomic approach was employed to analyze the metabolic consequences of MTX (0, 10, 40, and 100 mg/kg) in the urine and liver of healthy rats (control diet) and in a model of NASH (methionine-choline deficient diet). Histopathological analysis confirmed baseline (0 mg/kg) liver necrosis, liver inflammation, and lipid accumulation in the NASH model. Administration of MTX (40 and 100 mg/kg) led to liver necrosis in the control cohort, whereas the NASH cohort also displayed biliary hyperplasia and liver fibrosis (100 mg/kg), providing evidence of the synergistic effect of MTX and NASH. The complementary hepatic and urinary metabolic phenotypes of the NASH model, at baseline, revealed perturbation of multiple metabolites associated with oxidative and energetic stress, and folate homeostasis. Administration of MTX in both diet cohorts showed dose-dependent metabolic consequences affecting gut microbial, energy, nucleobase, nucleoside, and folate metabolism. Furthermore, a unique panel of metabolic changes reflective of the synergistic effect of MTX and NASH was identified, including the elevation of hepatic phenylalanine, urocanate, acetate, and both urinary and hepatic formiminoglutamic acid. This systems level metabonomic analysis of the hepatotoxicity of MTX in the context of NASH provided novel mechanistic insight of potential wider clinical relevance for further understanding the role of liver pathology as a risk factor for ADRs.

Adverse drug reactions (ADRs) are estimated to be responsible for approximately 7% of hospital admissions in the USA and UK (Lazarou *et al.*, 1998; Pirmohamed *et al.*, 2004). Methotrexate (MTX), a folate analog originally designed to inhibit dihydrofolate reductase for leukaemia treatment (Farber *et al.*, 1956), can cause ADRs including nephrotoxicity and hepatotoxicity (West, 1997; Widemann and Adamson, 2006). The risk of MTX-induced hepatotoxicity has been shown to increase in the presence of conditions such as diabetes mellitus or non-alcoholic steatohepatitis (NASH), with MTX alone also reported to cause a NASH-like injury (Langman *et al.*, 2001; Rosenberg *et al.*, 2007).

NASH is an advanced form of non-alcoholic fatty liver disease characterized by inflammation, macrovesicular steatosis, oxidative stress, and insulin resistance (Brunt, 2004). It is estimated to affect approximately 12% of USA adults, and approximately 20% of NASH sufferers develop liver cirrhosis (Williams *et al.*, 2011). The development of NASH is believed to involve a first hit, which is the result of imbalanced fat metabolism, and a second hit that involves metabolic and oxidative stress (Day and James, 1998). NASH has also been shown to influence hepatic drug metabolism by affecting CYP 450 enzyme activity (Fisher *et al.*, 2009) and ATP-binding cassette transporters (Hardwick *et al.*, 2011) and could therefore increase the risk of ADR occurrence.

NASH is modeled *in vivo* using several methods that include the use of specific diets or genetic models (Larter and Yeh, 2008). The methionine-choline deficient (MCD) diet has been widely employed to model NASH as it leads to a condition characterized by steatosis, mitochondrial dysfunction, inflammation, and oxidative stress (Rinella *et al.*, 2008; Rizki *et al.*, 2006). Moreover, increased CYP 2E1 expression (Leclercq *et al.*, 2000) and downregulation of stearoyl-CoA desaturase-1 (SCD-1) (Rizki *et al.*, 2006) have also been reported, all of which reflect the clinical NASH phenotype. However, the MCD model fails to recapitulate the metabolic syndrome component of NASH as it does not induce obesity, insulin resistance, or hyperglycemia (Larter and Yeh, 2008).

Metabonomics is a top-down untargeted systems approach that has been widely applied to characterize metabolic profiles of biofluids or tissues in disease states or in response to toxic interventions (Nicholson *et al.*, 1999). Metabonomic analysis typically relies on nuclear magnetic resonance (NMR) spectroscopy and mass spectrometry to generate high-resolution metabolic profiles. Statistical pattern recognition tools including principal components analysis (PCA) and orthogonal partial least squares discriminant analysis (O-PLS-DA) are applied to model the multivariate data (Fonville *et al.*, 2010). Metabonomics has been previously employed to investigate the serum metabolic effects of MTX in rheumatoid arthritis patients, where endogenous metabolic markers capable of predicting response to MTX treatment were identified, which included amino acids, taurine, and uracil (Wang *et al.*, 2012). It has also been applied to characterize the clinical NASH lipidomic plasma signature (Puri *et al.*, 2009), together with the serum metabolic phenotype of the MCD model of NASH in mice (Tanaka *et al.*, 2012), which revealed disruption of phospholipid and bile acid homeostasis.

In the present study, we employed a metabonomic approach to investigate the dose and time-dependent systemic endogenous and xenobiotic metabolic phenotype of MTX in the urine and liver of healthy animals and in the context of NASH, as modeled by the MCD diet.

## MATERIALS AND METHODS

### 

#### 

##### Animal husbandry and MTX administration

This study was approved by the Institutional Animal Care and Use Committee (IACUC) at the University of Arizona, and is in accordance with NIH guidelines for the care and use of experimental animals. Male Sprague Dawley rats (weighing 200–250 g prior to the beginning of the experiment, *n* = 44; Harlan Laboratories, Indianapolis, IN, USA) were randomly split into two groups (*n* = 22) which were either fed *ad libitum* a choline sufficient and amino-acid defined diet or the equivalent methionine-choline deficient diet (Dyets Inc, Bethlehem, PA) and tap water, for a period of 8 weeks. The rats were acclimated to 12-hour light and dark cycles in an AAALAC-accredited animal facility for 1 week prior to the initiation of experiments. The rats in each diet group were then randomly assigned to be administered 10, 40, and 100 mg/kg of MTX dissolved in 0.3-M sodium bicarbonate (Toronto Chemicals, Toronto, Canada) or vehicle (0.3 M sodium bicarbonate) via a single intra-peritoneal injection.

##### Sample collection

Urine samples were collected at 0 h (pre-dose, −6 to 0-hour collection; 0 h) and across the following time periods 6–12 (12 h), 18–24 (24 h), and 36–48 (48 h) hours post-dose via metabolism cages into 50-ml tubes on ice containing sodium azide (1 ml, 1% w/v in water). At each time point, the urine was transferred to a clean 50-ml conical tube and immediately stored at −80°C. At 96-hours post-dose (96 h), animals were euthanized via CO_2_ asphyxiation and livers were immediately snap-frozen in liquid nitrogen and stored at −80°C until analysis.

##### Liver histopathology and imaging

Liver tissue for histomorphologic examination was taken from the medial lobe of each animal, fixed in 10% neutral-buffered formalin for 24 h, then placed in 70% ethanol until paraffin embedding and hematoxylin and eosin (H&E) staining. Masson Trichrome staining was also performed using the Masson Trichrome Kit from Sigma-Aldrich (St. Louis, MO) per manufacturer's instructions. All slides were imaged with a Leica DM4000B microscope, DFC450 camera, and Leica Application Suite software (Leica Microsystems, Wetzlar, Germany). Sections were examined by light microscopy by a board certified blinded veterinary pathologist (Burkhardt *et al.*, 2011). Liver necrosis, inflammation, fibrosis, lipid accumulation, and biliary hyperplasia were scored. The scoring criteria were as follows: 0 (no lesion), 1 (<10% lesion; minimal), 2 (10–25% lesion; mild), 3 (25–40% lesion; moderate), 4 (40–50% lesion; marked), and 5 (>50% lesion; severe). Representative histology images were acquired with a Leica DM4000B microscope, DFC450 camera, and Leica Application Suite software (Leica Microsystems).

##### Sample preparation and ^1^H-NMR spectroscopy of hepatic aqueous-soluble extracts

Liver metabolite extraction and ^1^H-NMR spectroscopy of the hepatic aqueous-soluble extracts was performed as previously described (Beckonert *et al.*, 2007). Briefly, liver tissue samples (mean 52.5 mg, STD ± 0.73) were added to acetonitrile/water (1.5 ml, 1:1). The samples were homogenized with zirconia beads in a homogenizer (Qiagen Tissue Lyser, Retsch GmBH, Haan, Germany) at 6500 Hz for two 45-s periods, with an intermediate 5-min cooling period on dry ice. The samples were then kept on ice for 45 min prior to centrifugation at 17,000 × g for 15 min at 4°C. The supernatants were concentrated and dried overnight in a centrifugal evaporator (SpeedVac, Thermoscientific, Waltham, MA) at room temperature. The dried supernatants were reconstituted in phosphate buffer (600 μl of a 0.2-M solution) containing 100% D_2_O, sodium azide (NaN_3_, 3mM), and 3-(trimethylsilyl)-[2,2,3,3–^2^H_4_]-propionic acid sodium salt (TSP; 1mM)), vortexed for 30 s and then centrifuged at 17,000 × g for 15 min at 4°C. The supernatants (550 μl) were transferred to 5-mm NMR tubes (NMR Precision tube 507-HP-7, Norell, Landisville, NJ). NMR spectral data were acquired on a Bruker Avance-600 spectrometer (Bruker Biopsin, Rheinstetten, Germany) operating at 600.13-MHz (14.1 T) ^1^H frequency and at a temperature of 300 K, using a Bruker TXI probe and an automated sample handling carousel (Bruker). A standard one-dimensional solvent suppression pulse sequence was used to acquire the free induction decay (FID; relaxation delay − 90° pulse − 4-μs delay − 90° pulse − mixing time − 90° pulse − acquire FID) (Beckonert *et al.*, 2007). For each experiment, 64 transients were collected into 64-k data points using a spectral width of 12,000 Hz, with a relaxation delay of 4 s, an acquisition time of 2.7 s, and a mixing time of 100 ms. A Carr-Purcell-Meiboom-Gill (CPMG) spin-echo pulse sequence, with a fixed spin-spin relaxation delay 2*nτ* of 240 ms (*n* = 300, *τ* = 400 μs), was also used to acquire the FID (relaxation delay − 90° pulse − (*t* − 180° − *t*)*_n_* − acquire FID) (Beckonert *et al.*, 2007). The CPMG pulse sequence is used to attenuate signals from large macromolecules such as lipids and proteins. Three hundred and eighty four transients were collected into 64-k data points using a spectral width of 12,000 Hz, with a relaxation delay of 4 s and an acquisition time of 2.7 s. The D_2_O present in the buffer provided a field frequency lock, whereas TSP served as the chemical shift reference. A line-broadening factor of 0.3 Hz was applied to all spectra prior to Fourier Transformation (FT).

##### ^1^H-NMR spectroscopy of urine

^1^H-NMR spectroscopy of urine was performed as previously described (Beckonert *et al.*, 2007). Briefly, urine was mixed with phosphate buffer (2:1; 600 μl total volume; 0.2 M as above) and vortexed for 1 min. The samples were then centrifuged at 17,000 × g for 15 min at 4°C. The resulting supernatants (550 μl) were then transferred to 5-mm NMR tubes (507-HP-7). NMR spectral data were acquired on a Bruker Avance-600 spectrometer operating at 600.13-MHz (14.1 T) ^1^H frequency and at a temperature of 300 K using a Bruker TXI probe and an automated sample handling carousel (Bruker). A standard one-dimensional solvent suppression pulse sequence was used to acquire the FID as described above. For each experiment, 320 transients were collected into 64-k data points using a spectral width of 12,000 Hz, with a relaxation delay of 4 s, an acquisition time of 2.7 s, and mixing time of 100 ms. A line-broadening factor of 0.3 Hz was applied to all spectra prior to FT.

The NMR spectra were initially processed using TopSpin 3.0 NMR software (Bruker), where they were manually phased, baseline adjusted, and referenced to the TSP resonance at 0 ppm. Full-resolution NMR data were imported into MATLAB (R2012, Mathworks Inc., Natick, MA), using an in-house script, for further processing. This included the removal of the TSP, MTX related and water resonances in the hepatic aqueous-soluble component spectra and the TSP, MTX related, urea and water resonances from the urine spectra. Probabilistic quotient normalization and spectral alignment were performed in each diet cohort data set separately, apart from when performing the diet baseline comparison (using an in-house script) (Dieterle *et al.*, 2006). Spectral assignments were performed using Statistical TOtal Correlation Spectroscopy (STOCSY) (Cloarec *et al.*, 2005), spectral databases (in-house, Human Metabolome Database and Biological Magnetic Resonance Bank), Chenomx NMR Suite (Chenomx, Edmonton, Alberta, Canada), and previously published assignments (Nicholson *et al.*, 1995; Waters *et al.*, 2005). The assignments of uracil, hypoxanthine, inosine, uridine, maltose, mannose, choline, phosphocholine, and glycine in the hepatic aqueous-soluble extracts and dimethylamine, methylamine, phenylacetylglycine, putrescine, 3-indoxylsulphate, tartrate, glucosan, and taurine in the urine were confirmed by addition of the pure standard compounds. Please note that glutathione refers to total glutathione (sum of oxidized and reduced glutathione). The CPMG spectra of the hepatic aqueous-soluble extracts were acquired and modeled in order to minimize the contribution from lipids that arose from the MCD diet cohort.

The urinary excretion of MTX following administration of 100 mg/kg in both diet cohorts was calculated by performing local baseline correction prior to integration of the MTX resonance at 8.63 ppm in TopSpin. The integral of TSP (which was of known concentration) and the excreted urine volumes per rat at each time point were used to calculate the urinary amount of MTX (in micrograms). The results were verified independently by mass spectrometry (*R* > 0.99).

##### Statistical analysis

Multivariate statistical tools were employed to analyze the ^1^H-NMR data from both the urine and hepatic extracts. PCA was initially applied to investigate the baseline diet comparison and the effect of MTX by characterizing the inherent clustering of the data and the presence of potential outliers (Fonville *et al.*, 2010). O-PLS-DA was subsequently performed, which discriminated the pre-dose and post-dose groups. O-PLS-DA filters out variation that is orthogonal to class membership and hence improves model interpretability. In order to avoid over-fitting the data, a 7-fold cross-validation was used and statistical parameters (R^2^Y and Q^2^Y representing the goodness of fit and predictive ability) were calculated (Fonville *et al.*, 2010). Permutation tests (*n* = 1000 permutations of the class membership) were also used to test the validity of each model. Heatmaps based on the Pearson correlation coefficient values (R) of the identified discriminatory resonances from the O-PLS-DA models were also constructed with a cut-off *R* value of |0.5|. Prism 5.0 (Graphpad; La Jolla, CA) was used for non-parametric univariate analysis (Kruskal-Wallis with Dunn's multiple correction test). A significance threshold value of p < 0.05 was set throughout.

## RESULTS

### 

#### Liver Histopathology

**FIG. 1.**
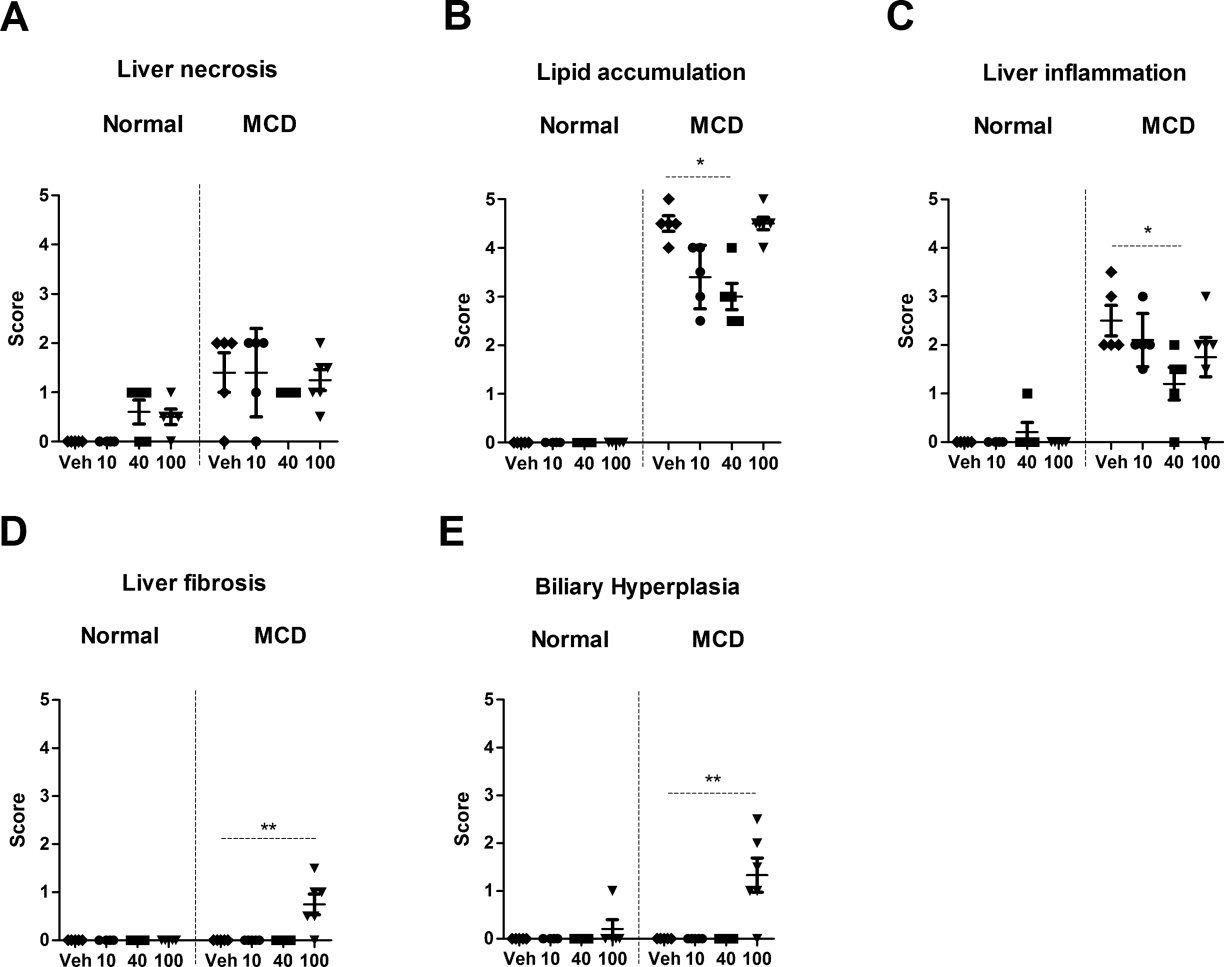
Histopathology scores representing liver necrosis (A), lipid accumulation (B), liver inflammation (C), liver fibrosis (D), and biliary hyperplasia (E) (average and standard deviation) following administration of MTX (vehicle, 10, 40, and 100 mg/kg) in both the control and MCD diet cohorts. Statistical significance is indicated by one or two asterisks representing p < 0.05 and p < 0.01, respectively. There are five animals per group apart from the 100-mg/kg dose in the MCD diet cohort which has six. The scoring criteria are: 0 = no significant lesions; 1 = minimal; 2 = mild; 3 = moderate; 4 = marked; and 5 = severe. Veh: vehicle.

**FIG. 2.**
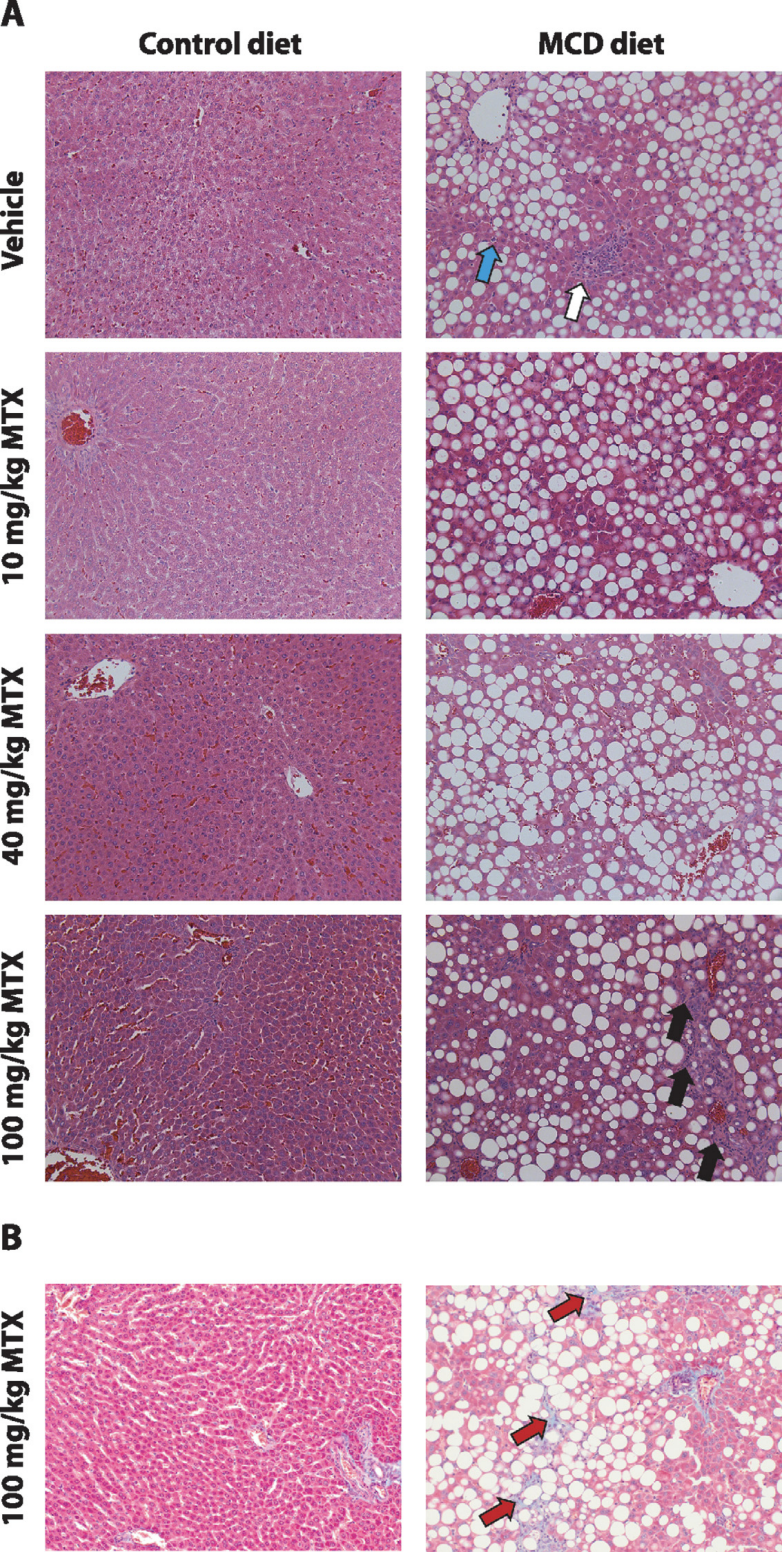
(A) Representative histology images of H&E-stained control and MCD liver sections following vehicle or 10, 40, or 100-mg/kg MTX and (B) Masson Trichrome stained liver sections following 100-mg/kg administration in both diets (×20 magnification). White and cyan arrows indicate inflammation and single cell necrosis in the MCD diet cohort baseline respectively. Black and red arrows indicate biliary hyperplasia and liver fibrosis, respectively, in the MCD cohort following administration of MTX (100 mg/kg).

Histopathological assessment of the control diet cohort revealed hepatic necrosis following MTX treatment (40 and 100 mg/kg; Figs. 1A and 2). In the control diet cohort, one animal also developed liver inflammation and another biliary hyperplasia following MTX treatment (40 and 100 mg/kg, respectively; Figs. 1C and E, respectively). In contrast, the MCD diet cohort had a background (vehicle group) characterized by liver necrosis, lipid accumulation, and liver inflammation (Figs. 1A–C, respectively, and Fig. 2). Following treatment of the MCD diet cohort with MTX (40 mg/kg), a statistically significant reduction in lipid accumulation and liver inflammation was observed (Figs. 1B and C, respectively, and Fig. 2). The MCD diet cohort rats also developed minimal liver fibrosis and mild biliary hyperplasia (black arrow, Fig. 2) following treatment with the highest dose of MTX (100 mg/kg; Figs. 1D and E, respectively).

Body weight was monitored at the beginning and end of the study. The average baseline weight of the control diet cohort animals was 390 g (STD ± 57 g) whereas that of the MCD diet cohort was 200 g (STD ± 46 g). In the control diet cohort, weight loss was observed following administration of MTX with an average percentage weight loss of 7%, 16%, and 26% for the cohorts administered 10, 40, and 100-mg/kg MTX, respectively. The MCD diet cohort also experienced weight loss with an average percentage weight loss of 18%, 20%, and 20% for the cohorts administered 10, 40, and 100-mg/kg MTX, respectively (Supplementary fig. 1).

#### Hepatic and Urinary Metabolic Phenotype of the NASH Model (MCD Diet Cohort Baseline)

**FIG. 3.**
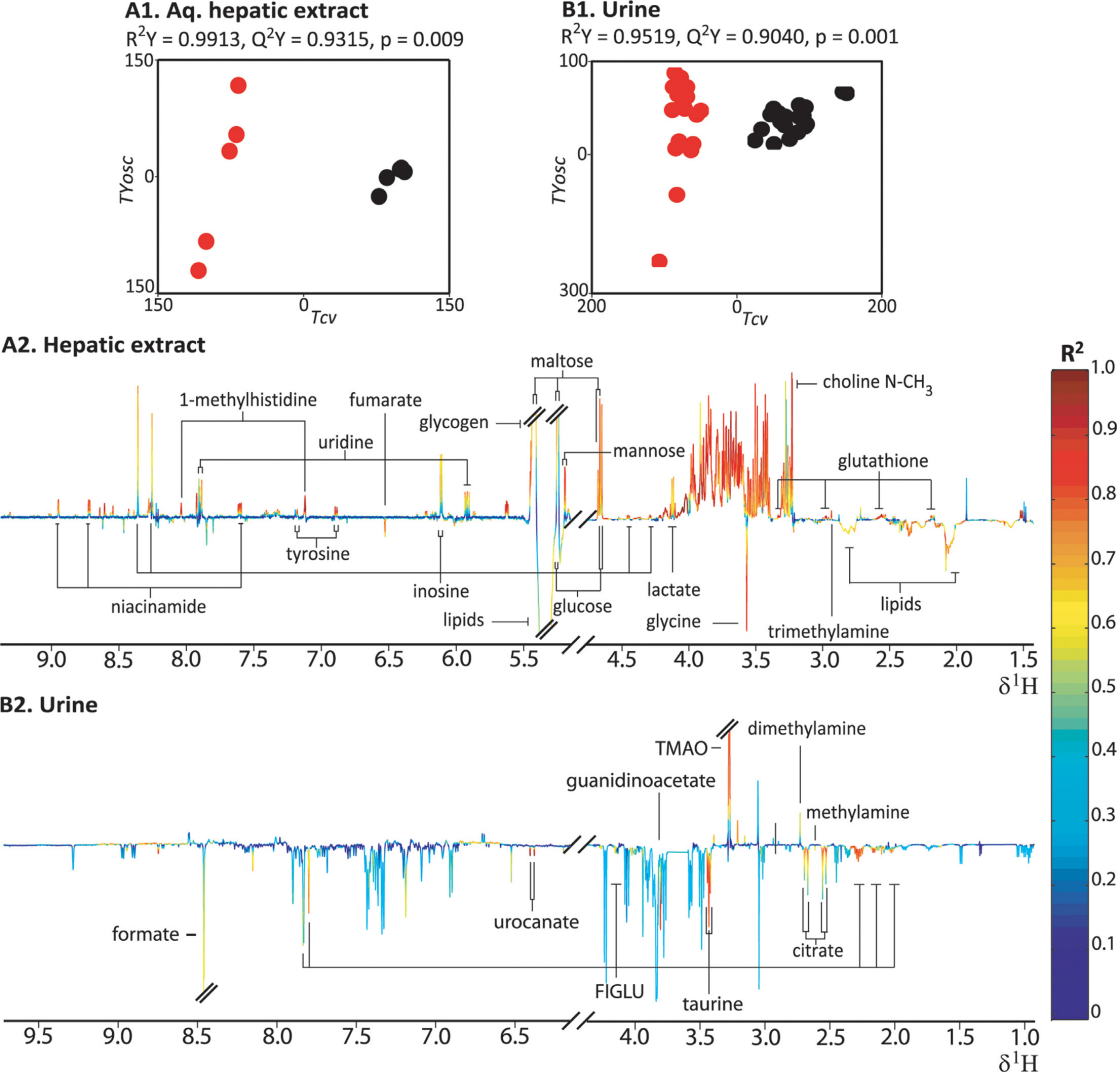
Hepatic aqueous-soluble extracts (A1, A2) and urinary (B1, B2) O-PLS-DA models distinguishing between the baseline of the control and MCD diet cohort. The scores plots (A1 and B1) depict the separation of the MCD (red; *n* = 5 in the liver and *n* = 20 in the urine) and control diet cohort (black; *n* = 5 in the liver and *n* = 20 in the urine) in multivariate space. Tcv is the predictive component, whereas TYosc is the orthogonal component. The loadings coefficient plots (A2 and B2) illustrate the discriminatory spectral resonances with the height of spectral peaks representing covariance. The color-scale corresponds to the coefficient of determination (R^2^) ranging from low (0; blue) to high (red; 1). Resonances pointing upward indicate an increase in that specific resonance in the control cohort and vice versa. FIGLU—formiminoglutamic acid; Choline-NMe_3_—NMe3 resonance of choline, phosphocholines and glycerophosphocholines; TMAO—trimethylamine-*N*-oxide.

Pair-wise O-PLS-DA models were computed to identify the discriminatory metabolites between the two diet cohorts for each biological matrix. A robust O-PLS-DA model (Fig. 3A; *R*^2^*Y* = 0.9913, *Q*^2^*Y* = 0.9315, and *p* = 0.009) revealed complete differentiation between the hepatic aqueous-soluble extract baseline (vehicle) metabolic profiles of the two diet cohorts as evidenced from the cross-validated predictive component of the scores plot (Tcv, cross-validated predictive scores; Fig. 3A1). The corresponding coefficient plot (Fig. 3A2) showed that the metabolic signature of the MCD diet cohort included depleted levels of glucose, glycogen, mannose, maltose, glutathione, lactate, niacinamide, choline, phosphocholine, trimethylamine, tyrosine, 1-methylhistidine, inosine, and uridine, together with elevated levels of glycine, lipids, fumarate, and formiminoglutamic acid (FIGLU), with respect to the baseline control diet cohort.

The O-PLS-DA model comparing the urinary baseline (pre-dose, 0 h) metabolic profiles of the two diet cohorts (Fig. 3B; R^2^Y = 0.9519, Q^2^Y = 0.9040, and p = 0.001) indicated complete discrimination as evidenced from the scores plot (Tcv; Fig. 3B1). The corresponding coefficient plot (Fig. 3B2) indicated that, relative to the control diet, the MCD diet cohorts had elevated levels of citrate, urocanate, guanidinoacetate, methylamine, taurine, formate, and FIGLU, and depleted levels of trimethylamine-*N*-oxide (TMAO) and dimethylamine.

#### Hepatic Metabolic Phenotype Following MTX Administration in the Control Diet Cohort

**FIG. 4.**
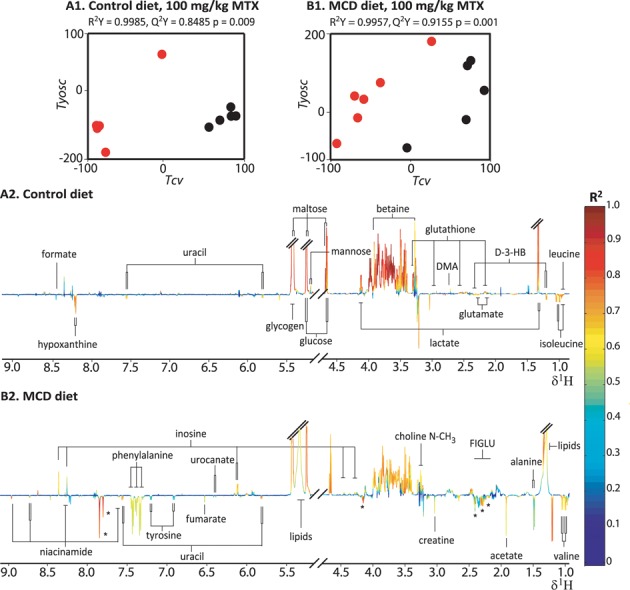
Hepatic extract O-PLS-DA models comparing the vehicle cohorts and 100-mg/kg MTX administration cohorts in the control diet (A1 and A2; *n* = 5 for the vehicle group and *n* = 5 for the MTX dosed group) and MCD diet (B1 and B2; *n* = 5 for the vehicle group and *n* = 6 for the MTX doses group). The score plots (A1 and B1) depict the separation of the MTX dose (red) and vehicle (black) cohorts in multivariate space. Key: See the legend of Fig. 3. DMA: dimethylamine; D-3-HB: D-3-hydroxybutyrate; Asterisks: FIGLU formiminoglutamic acid.

O-PLS-DA pair-wise models were employed to characterize the metabolic consequences of MTX administration on the hepatic aqueous-soluble extract metabolic profiles of the control diet cohort, relative to the vehicle cohort. The statistically robust O-PLS-DA model characterizing the effect of 100-mg/kg MTX administration (Fig. 4A; R^2^Y = 0.9985, Q^2^*Y* = 0.8485, and p *=* 0.009) achieved complete separation between the two groups in the scores plot (Tcv; Fig. 4A1). The corresponding coefficient plot (Fig. 4A2) revealed that the administration of 100-mg/kg MTX led to the depletion of lactate, glucose, maltose, mannose, glycogen, betaine, inosine, dimethylamine, and glutathione. It also led to the concomitant rise in the levels of valine, glutamate, leucine, isoleucine, glycerophosphocholine, D-3-hydroxybutyrate, formate, uracil, hypoxanthine, creatine, and choline following MTX administration relative to the vehicle cohort.

**FIG. 5.**
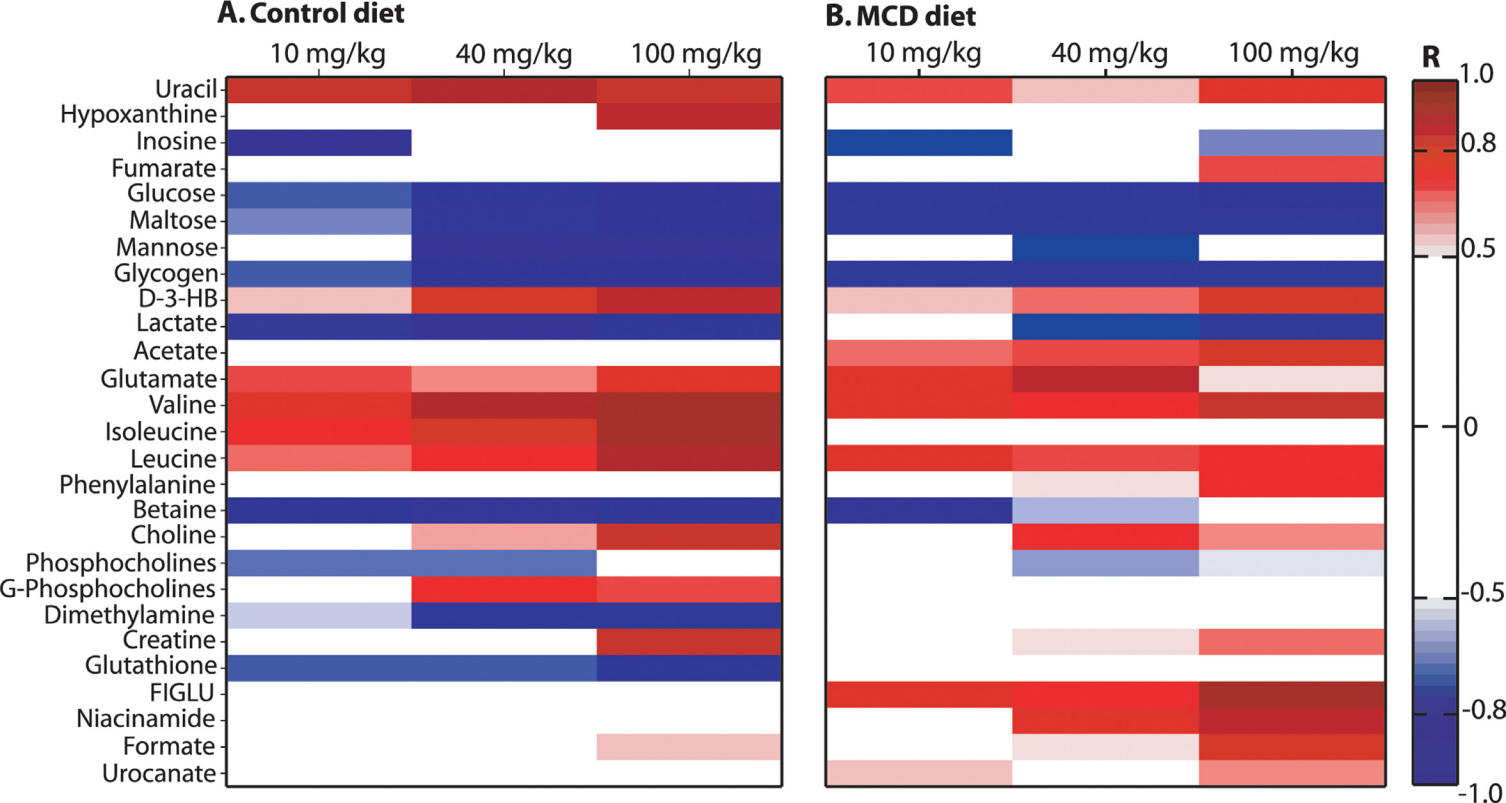
Heatmap describing the hepatic metabolic effects of MTX administration in the control diet (A) and MCD diet (B). The heatmap was constructed using the maximum Pearson correlation coefficient values (*R*) for each metabolite from the hepatic extract O-PLS-DA models distinguishing the metabolic profiles of MTX treated (10, 40, 100 mg/kg) and the vehicle cohort, in the control diet (A) and MCD diet (B). The scale shown on the right has a range from −1 (perfect negative correlation; blue color) to 1 perfect positive correlation; red color), whereas a cut-off value of |0.5| was set. Key: See the legend of Fig. 4.

The O-PLS-DA models comparing the hepatic metabolic profiles of the vehicle with the 10- and 40-mg/kg MTX administration groups (Supplementary fig. 2) were statistically robust and identified a panel of discriminatory metabolites for each dose group, including an elevation of glutamate, valine, isoleucine, leucine, and uracil, together with depletion of betaine, glutathione, dimethylamine, glycogen, and glucose, relative to the control cohort. The scale of metabolic perturbations induced by MTX administration was progressive and was maximal at the highest dose (100 mg/kg). Hepatic changes reflective of a dose response included elevation of valine, isoleucine, leucine and depletion of glucose, dimethylamine, glycogen, and glutathione. Furthermore, a number of unique metabolic changes were observed specific to the administration of 100-mg/kg MTX relative to the vehicle, which included increased levels of hypoxanthine, formate, and creatine. A summary of the hepatic metabolic effects induced by MTX administration at all doses in the control diet cohort is presented in Fig. 5A in the form of a heatmap, which displays the given *R* for each discriminatory metabolite obtained from the O-PLS-DA coefficients.

***Hepatic Metabolic Phenotype Following MTX Administration in the MCD Diet***

O-PLS-DA pair-wise models were computed to identify the discriminatory metabolites related to the effect of MTX administration at each dose level (10 mg/kg, 40 mg/kg, and 100 mg/kg) relative to the vehicle group in the MCD diet cohort. The statistically robust O-PLS-DA model of the vehicle and 100-mg/kg MTX administration group of the MCD diet cohort (Fig. 4B; R^2^Y= 0.9957, Q^2^Y= 0.9155, and p= 0.001) differentiated completely between the two groups (Tcv; Fig. 4B1). The corresponding coefficient plot (Fig. 4B2) indicated that the administration of 100-mg/kg MTX led to the rise of the levels of uracil, fumarate, D-3-hydroxybutyrate, valine, leucine, phenylalanine, glutamate, creatine, choline, urocanate, FIGLU, formate, and niacinamide, whereas inosine, phosphocholine, lactate, glycogen, maltose, and glucose were depleted. The increase in fumarate was unique to the model characterizing the effect of high dose MTX administration (100 mg/kg) in the MCD diet cohort. Significant hepatic metabolic perturbations were also identified following administration of both 10- and 40-mg/kg MTX including the depletion of glucose, maltose, and glycogen, and the elevation of leucine, isoleucine, glutamate, uracil, and FIGLU. The scale of metabolic change was reflective of the dose response with respect to an increase in phenylalanine, acetate, glutamate, and FIGLU and decrease in lactate levels. A summary of the hepatic metabolic effects induced by MTX administration at all doses for the MCD diet cohort is presented in Fig. 5B. Elevation of phenylalanine was also observed to be statistically significant in a regression analysis of the hepatic aqueous-soluble extract metabolic profiles from both diet cohorts with the biliary hyperplasia and fibrosis scores (R^2^Y= 0.5218, Q^2^Y= 0.2423, and p= 0.020, and R^2^Y= 0.5406, Q^2^Y= 0.2542 and p= 0.014, respectively). Integration of the phenylalanine resonance showed a statistically significant elevation following administration of 100-mg/kg MTX in the MCD diet cohort, when compared with the vehicle cohort, confirming the O-PLS regression results (p= 0.02; Supplementary fig. 3).

#### Urinary Metabolic Phenotype Following MTX Administration in the Control Diet

The collection of urine samples at sequential time points facilitated the characterization of the temporal metabolic consequences of MTX at each dose level for each diet cohort. O-PLS-DA pair-wise models were constructed to investigate the differentiation of pre-dose urinary baseline metabolic profiles (0 h) and post-treatment metabolic profiles (12 h, 24 h, and 48 h). The O-PLS-DA models that discriminated the metabolic effect of the 100-mg/kg dose of MTX from baseline had the highest statistical significance and greatest predictive ability and will be further discussed.

**FIG. 6.**
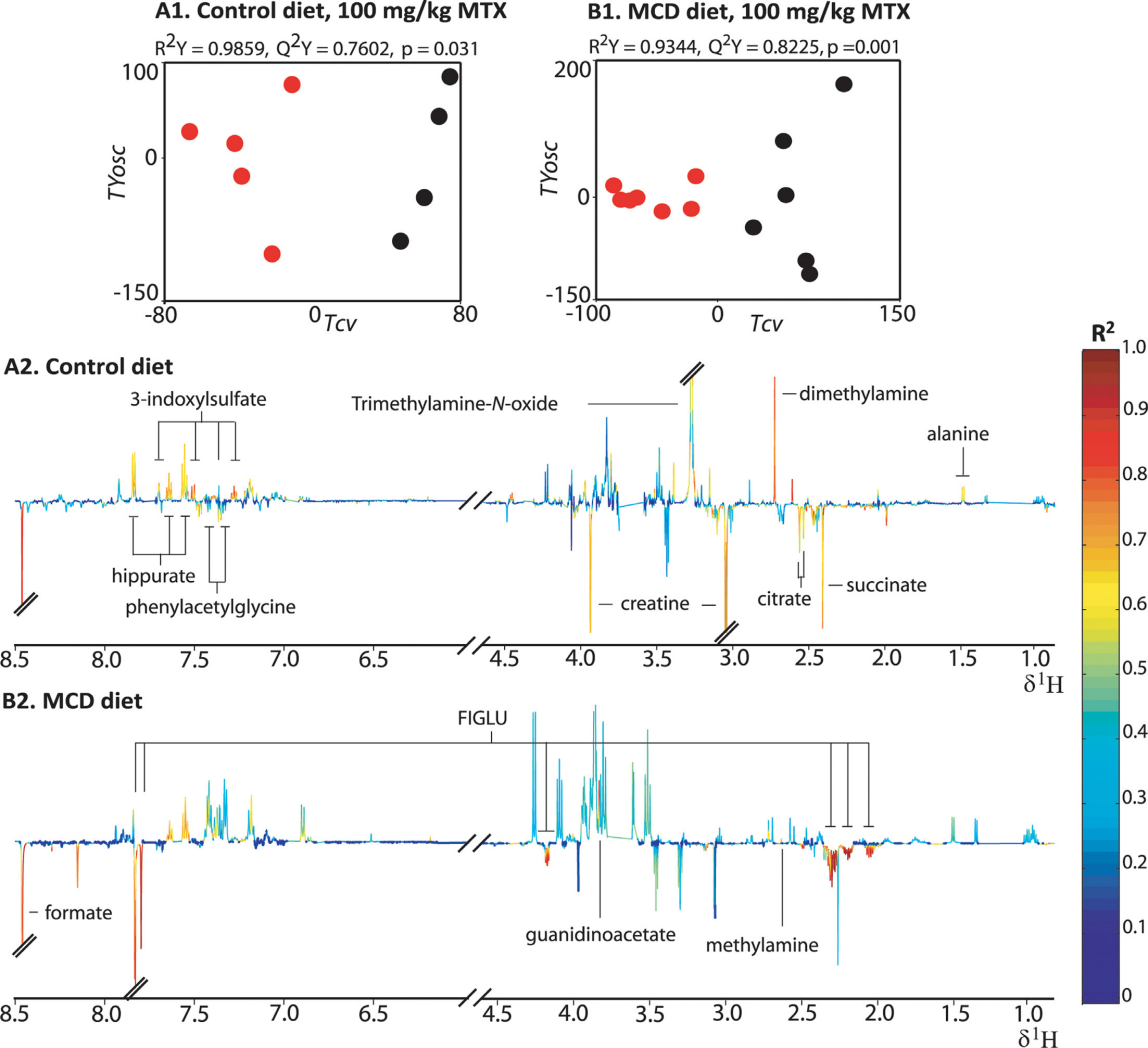
Urinary O-PLS-DA models comparing the pre-dose time point and the 48-h post-dose time point after 100-mg/kg MTX administration in the normal diet (A1 and A2; *n* = 4 at 0 h and *n* = 5 at 48 h) and MCD diet (B1 and B2; *n* = 6 at 0 h and *n* = 7 at 48 h). Key: See the legend of Fig. 3.

The O-PLS-DA models characterizing the comparison of the urine pre-dose (0 h) metabolic profiles with each of the post-dose (100-mg/kg MTX) time points (12 h, 24 h, and 48 h) in the control diet cohort showed that as time progressed, the effect of MTX became more pronounced (for a summary of the statistical parameters for each model please see Supplementary table 1). The statistically robust model comparing the metabolic profiles of the control diet 0-h and 48-h time points (Fig. 6A; R^2^Y = 0.9859, Q^2^Y = 0.7608, and p = 0.034) achieved complete separation between the two groups in the scores plot (Tcv; Fig. 6A1). The corresponding coefficient plot (Fig. 6A2) revealed a depletion of alanine, methylamine, dimethylamine, hippurate, TMAO, and 3-indoxylsulfate, together with an elevation of the levels of succinate, creatine, phenylacetylglycine, and formate.

#### Urinary Metabolic Phenotype Following MTX Administration in the MCD Diet

The corresponding urinary analysis of the metabolic effect of MTX administration (100 mg/kg) in the MCD diet cohort also indicated a time-dependent progressive effect of MTX that was maximal at 48 h post-treatment (Supplementary table 1 provides a summary of the statistical parameters of the models). The statistically robust model comparing the metabolic profiles of the MCD diet 0-h and 48-h time points (Fig. 6B; R^2^Y = 0.9985, Q^2^Y = 0.8485, and p = 0.001) achieved complete differentiation between the two groups in the scores plot (Tcv; Fig. 6B1). The equivalent coefficient plot (Fig. 6B2) indicated that MTX administration led to an elevation in the levels of FIGLU and formate, and the depletion of methylamine, alanine, and hippurate.

Following administration of MTX (100 mg/kg), the urinary metabolic phenotype showed a progressive depletion from 12 h to 48 h of methylamine and hippurate and elevation of formate and FIGLU. The depletion of alanine at 48 h was a unique metabolic effect that was specific for this time point. Depletions in the levels of 3-indoxylsulfate and phenylacetylglycine were also observed but these were restricted to the 24-h time point, whereas TMAO was depleted in both 12 h and 24 h but had returned to baseline levels by 48 h. With respect to the models characterizing the metabolic effect of the 10-mg/kg and 40-mg/kg doses of MTX, elevation of FIGLU and formate was also observed at 48 h post-dose. However, at that time point, both of the lower doses of MTX also led to the depletion of methylamine and 3-indoxylsulfate, together with the elevation of dimethylamine, all of which were not observed at the corresponding time point after 100-mg/kg MTX administration.

**FIG. 7.**
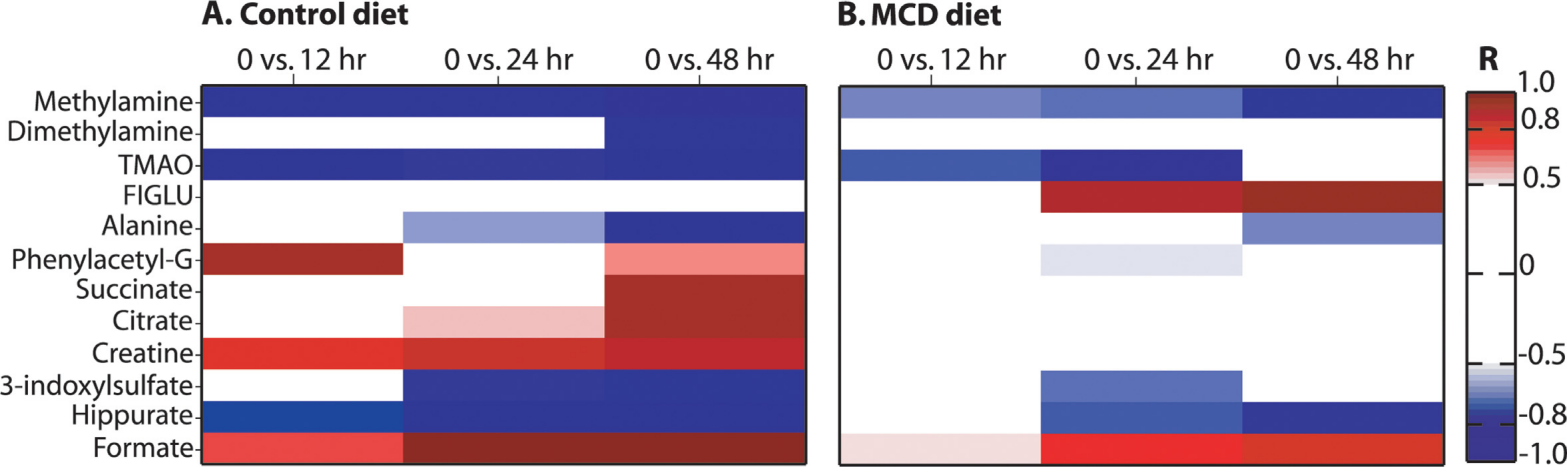
Heatmap describing the urinary metabolic effects of MTX administration in the control diet (A) and MCD diet (B). Based on O-PLS-DA models that discriminated between the metabolic profiles of the post-dose time points (12 h, 24 h, and 48 h) of MTX administration (100 mg/kg) and the pre-dose time point (0 h), in both diets. Key:See the legend of Fig.3. Phenylacetyl-G: phenylacetylglycine.

The comparison of the effects of administration of MTX (100 mg/kg) in the two diet cohorts showed several diet specific MTX-induced metabolic effects. At 48 h post-dose, elevations of phenylacetylglycine, creatine, citrate, and succinate were specific only to the control diet cohort, whereas elevated levels of FIGLU were specific only to the MCD diet cohort (Fig. 7 illustrates a heatmap summary of the urinary metabolic effect of MTX administration (100 mg/kg) in both diet cohorts).

Finally, temporal urinary levels of the MTX parent compound following administration of the 100-mg/kg dose were also compared between the two diet cohorts at the 12-h, 24-h, and 48-h time points. The levels of MTX were greater in the control diet cohort than the MCD cohort at all time points, reaching statistical significance at the 48-h time point (*p* = 0.0025; Supplementary fig. 4).

## DISCUSSION

### 

#### Characterization of the MCD Diet Model of NASH

The MCD diet had liver histopathology consistent with a phenotype reflective of clinical NASH (Brunt, 2004). Metabonomic analysis of the hepatic extracts and urine from the MCD diet cohort indicated a general impairment of energy and choline metabolism, coupled with glutathione depletion. The depletion of energy metabolites such as hepatic glucose and glycogen, relative to the control diet cohort, is indicative of energetic stress and could be related to the previously reported NASH-induced mitochondrial stress (Perez-Carreras *et al.*, 2003) or the inhibition of SCD-1 (Rizki *et al.*, 2006). Glutathione depletion is a marker of oxidative stress which is also characteristic of the NASH background (Merrell and Cherrington, 2011) and whose hepatic depletion was also potentially exacerbated by the methionine deficient nature of the MCD diet. Finally, the choline deficient nature of the diet was also likely to be directly linked to the depletion of choline-related metabolites such as hepatic trimethylamine and choline and urinary TMAO.

**FIG. 8.**
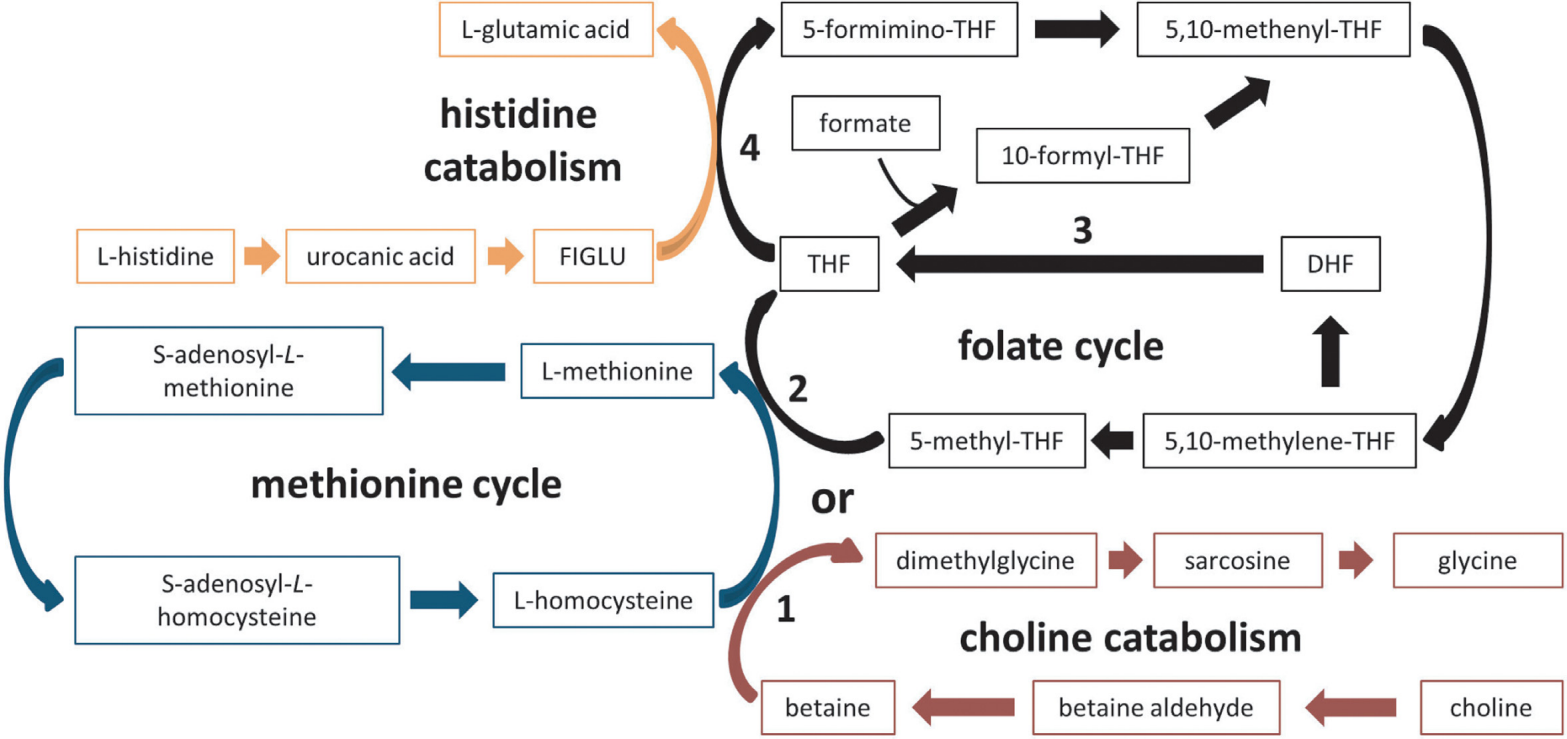
A simplified diagrammatic representation of the interaction of the methionine cycle (blue), folate cycle (black), choline catabolism (red), and histidine catabolism (orange) pathways is depicted above. (1) reveals where the choline catabolism pathway interacts with the methionine cycle, (2) indicates the point at which the methionine and folate cycles interact, (3) shows the metabolic reaction inhibited by methotrexate, and (4) indicates how the folate cycle can directly influence the histidine catabolism pathway. THF: tetrahydroflate; DHF: dihydrofolate; FIGLU: formiminoglutamic acid.

The choline and methionine deficient nature of this dietary model would have also directly impaired the folate cycle and one-carbon metabolism (Fig. 8). Impairment of the folate cycle would lead to reduced tetrahydrofolate (THF) production, which may explain the observed accumulation of urinary and hepatic FIGLU and urocanate and urinary formate. THF is required for the conversion of FIGLU to glutamate and FIGLU is a previously described urinary marker of folate deficiency (Rabinowitz and Tabor, 1958). Accumulation of urinary formate would arise from THF depletion and the subsequent impairment of formyl-THF production. Finally, the depleted levels of hepatic inosine and the elevated levels of hepatic glycine and urinary guanidinoacetate (a glycine metabolite) may also be linked to a general inhibition of the folate cycle (Fowler, 2001).

It is important to be aware of the limitations associated with the MCD diet as a model of NASH. Creation of a model that accurately reflects the clinical condition of NASH has proven difficult, because it would require replication of both the liver pathology and the systemic metabolic environment (Larter and Yeh, 2008). The MCD diet is believed to induce steatohepatitis and oxidative stress through the inhibition of phosphatidylcholine synthesis and impairment of hepatic lipid transport (Rinella *et al.*, 2008), but it is not thought to be an accurate reflection of the metabolic syndrome component of NASH because it does not induce obesity, insulin resistance, or hyperglycemia. Furthermore, the MCD diet induces significant weight loss as was also observed in our study (Larter and Yeh, 2008; Rinella *et al.*, 2008).

#### Effect of MTX in the Context of the Control Diet Cohort

Minimal liver necrosis was observed following administration of MTX (40 and 100 mg/kg) in the control diet cohort. Metabonomic analysis showed dose-related metabolic consequences in both sample matrices, together with a temporal effect in the case of urinary metabolic profiles.

The MTX-induced elevation of the levels of hepatic uracil is potentially due to the inhibition of thymidylate synthetase, whereas the observed depletion of hepatic inosine could result from the inhibition of phosphoribosylaminoimidazolecarboxamide formyltransferase (Genestier *et al.*, 2000). MTX could also affect the levels of nucleobases and nucleosides indirectly through the disruption of the folate cycle and its inhibition of THF formation, which could have other downstream consequences such as the observed elevation of urinary formate.

The observed effect of MTX, consistent at all doses, on hepatic energy metabolism, including the depletion of lactate, glucose, and glycogen, could be attributed to the previously reported inhibition of oxidative phosphorylation, which would lead to a higher utilization of glucose through other pathways (Yamamoto *et al.*, 1988). Furthermore, the depletion of the aforementioned metabolites could lead to lipid breakdown, as indicated by the elevated levels of hepatic D-3-hydroxybutyrate. This observed disturbance of energy metabolism could also be linked to a reduced caloric intake through the previously reported MTX-induced weight loss, which is linked to a disruption of gut absorption and mucositis (Naruhashi *et al.*, 2000). A reduction of caloric intake could also be linked to the observed depletion of hepatic glutathione (Szkudelski *et al.*, 2004) and choline-related moieties, such as hepatic phosphocholine and dimethylamine. However, a previous metabolic profiling investigation of caloric restriction observed a different panel of discriminatory urinary metabolites to that of our investigation (Connor *et al.*, 2004). Nevertheless, it is recommended that future studies should also record food consumption and employ an experimental design that would incorporate a pair feeding system in order to be able to easily identify any metabolic changes linked to fasting.

#### Effect of MTX in the Context of the NASH Background (MCD Diet Model)

We hypothesized that the presence of the NASH background, which can induce changes in drug metabolizing enzymes and hepatic transporters (Fisher *et al.*, 2009; Hardwick *et al.*, 2011), would alter MTX-induced metabolic consequences and toxic outcome. The administration of MTX (40 mg/kg) in the context of NASH resulted in a statistically significant histomorphologic reduction of lipid accumulation and inflammation. These observed reductions are suggestive of a protective effect due to the anti-inflammatory properties of MTX, whereas MTX has previously been reported to affect cholesterol transport (Coomes *et al.*, 2011). However, administration of the highest dose caused minimal liver fibrosis and biliary hyperplasia suggesting enhanced toxicity of MTX in the context of NASH.

Metabonomic analysis of the hepatic aqueous-soluble extracts revealed that, in part, the metabolic effect of MTX in the NASH model had a similar progressive pattern to the control diet cohort. Interestingly, MTX-induced metabolic effects specific to the NASH model were also observed, suggesting that the NASH baseline influenced the metabolic consequences of MTX administration. These included increased levels of hepatic FIGLU, urocanate, phenylalanine, acetate, niacinamide, and fumarate. The progressive dose-dependent elevations of fumarate, phenylalanine, and niacinamide did not follow the same pattern as the observed weight loss in the MCD diet cohort and therefore are more likely to be mechanistically indicative of MTX toxicity and not a direct consequence of either MTX or MCD-diet-induced weight loss. The elevations of hepatic metabolites associated with the folate cycle, such as FIGLU and urocanate, following MTX administration in the NASH model are signs of a significant inhibition of the folate cycle, greater than that observed in the MCD diet at baseline and greater than that observed after MTX administration in the control diet cohort. This combined effect of MTX and NASH, coupled to previous clinical reports of NASH-induced methionine cycle disruption triggered through glutathione depletion (Kalhan *et al.*, 2011), suggests a clinically relevant synergistic effect of NASH and MTX on folate metabolism (Fig. 8).

Hepatic phenylalanine was also identified by regression analyses as the sole endogenous metabolite associated with the development of biliary hyperplasia and liver fibrosis. Levels of hepatic phenylalanine are used as a liver function test because they are related to the activity of the phenylalanine hydroxylase enzyme, an important enzyme for phenylalanine degradation (Burke *et al.*, 1997). Higher levels of serum phenylalanine have been previously reported clinically following treatment with MTX and were linked to the inhibition of dihydropteridine reductase (Hilton *et al.*, 1989). However, the observed elevation of hepatic phenylalanine in our study is likely to be influenced by additional mechanisms because the levels of hepatic tyrosine remained unperturbed. Furthermore, the levels of urinary phenylacetylglycine following administration of 100-mg/kg MTX in the MCD diet cohort were also largely unperturbed during the time course of the study.

The parallel study of the xenobiotic metabolic profile enabled characterization of the effect of NASH on the disposition of MTX and showed increased urinary excretion of MTX in the control cohort at the 12-h, 24-h, and 48-h time points. This is explained by the greater urinary excretion of MTX that was observed for the MCD diet cohort relative to the control diet cohort within the first 6 h post-dose (Hardwick *et al.*, in preparation). The observed higher early urinary excretion of MTX in the MCD diet cohort could be due to the reported downregulation of canalicular transporters in the NASH model, and the relatively lower quantity of MTX in the feces, which indicate a shift in the excretion pathway of MTX toward urinary excretion (Hardwick *et al.*, in preparation). Furthermore, a previous study has also reported the downregulation of uptake transporter genes in disease-compromised livers as a hepatoprotective mechanism, indicating another potential mechanism behind the observed difference of urinary MTX excretion (Lake *et al.*, 2011).

#### Effect of MTX on Gut Microbial Co-metabolites

High dose MTX can cause intestinal toxicity and it has also been shown to affect the intestinal microbial composition (Naruhashi *et al.*, 2000). Both MTX (100 mg/kg) diet cohorts displayed mild inflammation and minimal apoptosis in the small intestine (Hardwick *et al.*, in preparation). Metabonomic analysis indicated that gut microbial co-metabolites, including urinary methylamine, TMAO, and 3-indoxylsulfate, were depleted in both diet cohorts following administration of 100-mg/kg MTX suggesting a potential perturbation of the gut microfloral balance (Nicholson *et al.*, 2012).

## SUMMARY

This systems level data analysis provided enhanced mechanistic phenotyping of a widely used model of NASH, revealing a metabolic phenotype characterized by energetic and oxidative stress, and further contextualized its application in experimental studies. Metabolic profiling of the hepatic and urinary dose and temporal response to MTX administration revealed a wide panel of metabolic consequences that provided novel mechanistic insight. Finally, the administration of MTX (100 mg/kg) in the context of NASH led to enhanced hepatotoxicity and the identification of a unique panel of metabolites which reflected this synergistic effect, providing mechanistic insight of potential translatable relevance for the clinical setting.

## SUPPLEMENTARY DATA

Supplementary data are available online at http://toxsci.oxfordjournals.org/.

## FUNDING

National Institute of Health (HD062489, ES006694, AI083927 to N.J.C.); Medical Research Council Integrative Toxicology Training Partnership (to M.K., M.C.). *Conflict of interest:* The authors state no conflict of interest and have received no payment in preparation of this manuscript..

## Supplementary Material

Supplementary Data
