# Supplementary material for: Systems Level Metabolic Phenotype of Methotrexate Administration in the Context of Non-alcoholic Steatohepatitis in the Rat
Source: Toxicol Sci. 2014 Aug 21;142(1):105–16. doi: 10.1093/toxsci/kfu160 (PMC4226764; doi:10.1093/toxsci/kfu160)
Supplement: Supplementary Data [file supp_142_1_105__index.html]

Systems Level Metabolic Phenotype of Methotrexate Administration in the Context of Non-alcoholic Steatohepatitis in the Rat — Supplementary Data 

# Systems Level Metabolic Phenotype of Methotrexate Administration in the Context of Non-alcoholic Steatohepatitis in the Rat

## Supplementary Data

**Files in this Data Supplement:**

- Supplementary Figure 1
- Supplementary Figure 2
- Supplementary Figure 3
- Supplementary Figure 4
- Supplementary Table 1
