# Supplementary figures and images for: Systems Level Metabolic Phenotype of Methotrexate Administration in the Context of Non-alcoholic Steatohepatitis in the Rat
Source: Toxicol Sci. 2014 Aug 21;142(1):105–16. doi: 10.1093/toxsci/kfu160 (PMC4226764; doi:10.1093/toxsci/kfu160)

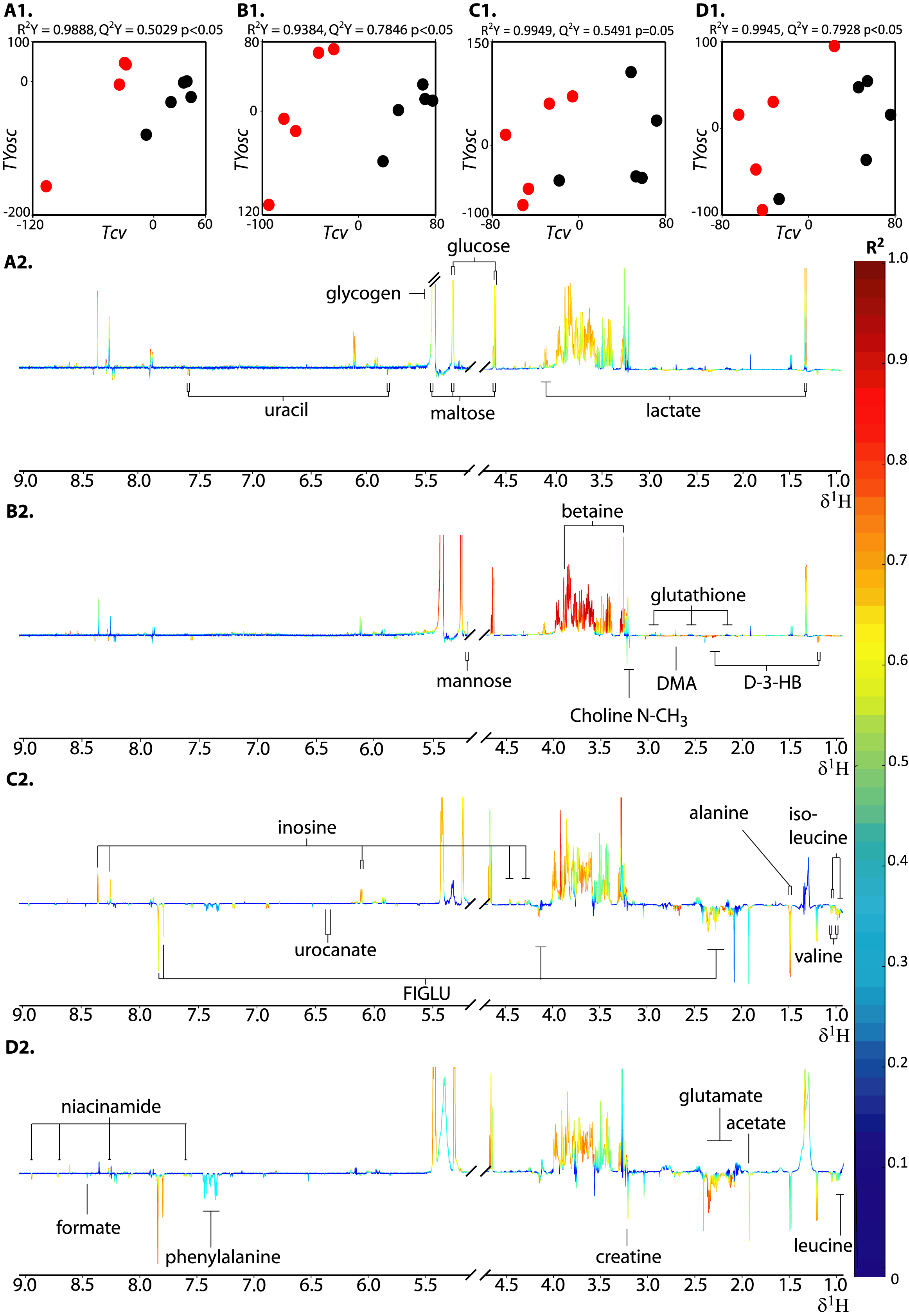

Supplement: Supplementary Data [file supp_kfu160_Kyriakides_et_al_supplementary_image_2.tif]
