# Supplementary material for: Systems Level Metabolic Phenotype of Methotrexate Administration in the Context of Non-alcoholic Steatohepatitis in the Rat
Source: Toxicol Sci. 2014 Aug 21;142(1):105–16. doi: 10.1093/toxsci/kfu160 (PMC4226764; doi:10.1093/toxsci/kfu160)
Supplement: Supplementary Data [file supp_kfu160_Supplementary_Table_1.docx]

| MTX dose | Comparison (model) | R^2^Y | Q^2^Y | Statistical sig. |
| --- | --- | --- | --- | --- |
| 100 mg/kg | 0 vs. 12 hours (control diet) | 0.9011 | 0.5907 | p < 0.05 |
|  | 0 vs. 24 hours (control diet) | 0.9404 | 0.7675 | p < 0.05 |
|  | 0 vs. 48 hours (control diet) | 0.9344 | 0.8225 | p < 0.05 |
| 100 mg/kg | 0 vs. 12 hours (MCD diet) | 0.9833 | 0.8135 | p < 0.05 |
|  | 0 vs. 24 hours (MCD diet) | 0.9958 | 0.7317 | p < 0.05 |
|  | 0 vs. 48 hours (MCD diet) | 0.9859 | 0.7602 | p < 0.05 |

Supplementary table 1: Summary of urinary O-PLS-DA models following administration of MTX (100 mg/kg) in both diet cohorts at the defined time collection periods.
